# The Anti-Inflammatory Effect of Lactose-Modified Hyaluronic Acid Molecules on Primary Bronchial Fibroblasts of Smokers

**DOI:** 10.3390/polym15071616

**Published:** 2023-03-24

**Authors:** Alice Donato, Federico Fontana, Rina Venerando, Antonino Di Stefano, Paola Brun

**Affiliations:** 1 Histology Unit, Department of Molecular Medicine, University of Padova, 35121 Padova, Italy; 2 Center for Nanomedicine and Tissue Engineering, A.S.S.T. Grande Ospedale Metropolitano Niguarda, Piazza dell’Ospedale Maggiore 3, 20162 Milan, Italy; 3 Department of Molecular Medicine, University of Padova, 35121 Padova, Italy; 4 Divisione di Pneumologia e Laboratorio di Citoimmunopatologia dell’Apparato Cardio Respiratorio, Istituti Clinici Scientifici Maugeri, IRCCS, 28013 Gattico-Veruno, Italy

**Keywords:** hyaluronic acid, inflammation, reactive oxygen species, Galectin 3, smoking, COPD, rehabilitation

## Abstract

The progression of smoking-related diseases is characterized by macrophage-mediated inflammation, which is responsible for an increased expression of proinflammatory cytokines and galectins, molecules that bind specifically to β-galactoside sugars. This study aimed to assess the anti-inflammatory and antioxidant effects of a broad selection of differently lactose-modified hyaluronic acids (HA) named HYLACH^®^, which are able to bind proinflammatory galectins. The best HYLACH ligands for Gal-3 were selected in silico and their activities were tested in vitro on primary human bronchial fibroblasts obtained from smokers and inflamed with the conditioned medium of activated U937 monocytes. Changes in cell viability, ROS generation, proinflammatory mediators, and MMP expression, at both gene and protein levels, were analyzed. The in silico results show that HYLACH with a percentage of lactosylation of 10–40% are the best ligands for Gal-3. The in vitro study revealed that HYLACH compounds with 10, 20, and 40% lactosylation (HYLACH-1-2-3) administrated to inflamed cell cultures counteracted the oxidative damage and restored gene and protein expression for *IL-1β*, *TNF-α*, *IL-6*, *Gal-1*, *Gal-3*, and *MMP-3* to near baseline values. The evidence that HYLACH attenuated macrophage-induced inflammation, inhibited MMP expression, and exhibited antioxidative effects provide an initial step toward the development of a therapeutic treatment suitable for smoking-related diseases.

## 1. Introduction

In smoking-related pathologies such as chronic obstructive pulmonary disease (COPD), a rather irreversible inflammatory disorder ranked number three in the world as a primary cause of death [1,2], the population of lung macrophages is expanded and correlates with the severity of inflammation [3]. These cells promote disease progression by releasing high levels of proinflammatory cytokines, driving the recruitment of neutrophils and monocytes to the lungs [4]. The major cytokines released by macrophages are interleukin-1β (IL-1β), tumor necrosis factor-α (TNF-α), and interleukin-6 (IL-6) and they are capable of stimulating bronchial cells to synthesize other proinflammatory molecules, free oxygen radicals (ROS), and MMPs [5,6]. Moreover, in vitro and in vivo studies also revealed that galectins, inflammatory molecules with a carbohydrate-recognition binding domain (CRD) for β-galactoside sugars, are overexpressed in chronic inflammatory diseases [7,8]. In particular, Galectin-1 (Gal-1) and Galectin-3 (Gal-3) act as regulators of inflammatory response in lung diseases such as COPD and idiopathic pulmonary fibrosis (IPF), making them good targets for drug design therapies [9,10,11]. Curiously, there is a singular lack of effective treatments in controlling COPD and lung fibrosis progression [12,13,14]. It is, therefore, necessary to study new treatments and explore new molecules capable of modulating the production of proinflammatory molecules.

Hyaluronic Acid (HA) is an extracellular matrix (ECM) molecule with anti-inflammatory properties that is currently used as an anti-inflammatory compound for many diseases [15,16]. Moreover, recent studies have demonstrated that glycosylated molecules significantly reduce in vitro and in vivo galectin expression and, consequently, inflammation [17,18] since the lactose residues can bind to the carbohydrate-recognition binding domain of galectins. It can be hypothesized that the combination of lactose with HA could reduce bronchial inflammation promoted by macrophages through the interaction with Galectin-3 (Gal-3) and that this reaction should be dependent upon the degree of HA lactosylation.

Computational simulations and in silico experimentation could be used to test the ability of molecules based on HA to interact with Gal-3, thus reducing the number of lead macromolecules. Indeed, the most promising target molecules are selected using molecular docking and molecular dynamics simulations. Computational modeling can screen the ligand that will presumably bind better to the target protein [19].

The first aim of this study was to test, in silico, the interaction between recently synthesized lactose-modified HA (HYLACH^®^) with different amounts of lactose-derived residues and the proinflammatory protein Gal-3.

The second aim was to evaluate the anti-inflammatory effect of the lead HYLACH molecules on an in vitro model of macrophage-mediated inflammation. For this purpose, we exposed monolayer-cultured primary bronchial fibroblasts obtained from smokers to a conditioned medium (CM) of U937 human-activated monocytes (macrophages) in the presence of HYLACH to assess changes in cell viability, in proinflammatory gene and protein expression, and intracellular ROS generation.

## 2. Materials and Methods

### 2.1. In Silico Methods

**Scheme 1:**
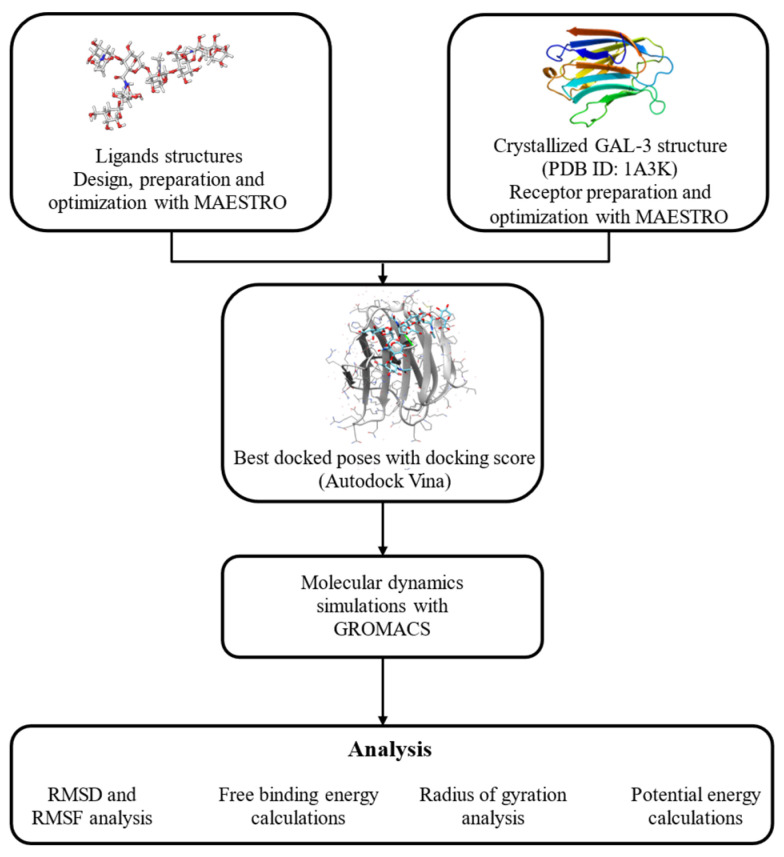
Flowchart of the workflow used for molecular docking and molecular dynamics simulations.

Scheme 1 includes a flowchart depicting the workflow used.

#### 2.1.1. Protein Retrieval and Protein and Ligand Preparation

The crystallographic structure of Gal-3 was downloaded from the Research Collaboratory for Structural Bioinformatics—Protein Data Bank (RCSB—PDB (PDB ID:1A3K)) (www.wwpdb.org, accessed on 14 February 2022). The initial configuration of the system was prepared by removing water molecules, ions, and cofactors and adding hydrogen atoms with MAESTRO software (version 2021-3) [20]. The structure of HA was designed using the 2D SKETCHER function present in MAESTRO considering the following structure: units of {β1→3} N-acetylglucosamine (GlcNAc) and {β1→4} glucuronic acid (GlcUA). Lactose was designed in the same way, consisting of a molecule of D-galactose and one of D-glucose joined by a glycosidic bond β{β1→β4}. The interactions between lactose and galactin-3 were evaluated using docking simulations to quantify the effects of the galactosylation of hyaluronic acid on galactin-3 binding.

#### 2.1.2. Molecular Docking

The molecular docking simulations with HA, lactose, and HYLACH at different percentages of lactosylation were performed using Auto-DockTools (Autodock Vina) [21], which employed an empirical scoring function based on the free energy of binding. All docking simulations were performed with a flexible ligand and a flexible receptor. For each ligand, different receptor-ligand poses were produced and the best pose (with a, therefore, more negative ΔG0) was used for the subsequent molecular dynamics simulations.

#### 2.1.3. Molecular Dynamics Simulations

The structures obtained from Autodock Vina were used as starting points for the molecular dynamics simulations. The parameterization of the ligand was performed through the online software PRODRG, which allowed the generation of the topology of the ligand [22]. All simulations and analyses were performed using Gromacs software (version 2022.3) (https://doi.org/10.5281/zenodo.5053220, accessed on 4 April 2022) using the AMBER force field [23]. The simulation box was prepared with PACKMOL [24]. The systems were solvated and neutralized by the addition of counterions. The minimization was performed using the steepest descent method with an equilibration procedure of 250 ns and several steps equal to 5000. The initial speeds used to equilibrate the system were obtained from a Maxwell distribution. The temperature was kept constant using a thermal bath at 300 K and with a coupling constant (τT) equal to 1.0 ps. The relative dielectric constant was set equal to 15. The cut-off radius was set at 0.8 nm for electrostatic interactions and at 1.2 nm for Van der Waals interactions. Finally, we proceeded with the second phase of equilibration conducted using the NPT ensemble and occurred in 250 ns (with a dt equal to 0.02). The cut-off radius was set at 1.0 nm for both electrostatic and Van der Waals interactions. The temperature was maintained at 300 K using a v-rescale thermometer and with a coupling constant (τT) of 1 ps [25]. Periodic boundary conditions were imposed and the pressure was maintained at 1 bar using the Berendsen barostat. The isothermal compressibility was set at 3 × 10^−4^ bar^−1^ and the coupling constant (τP) was 1 ps. Finally, we proceeded with the production phase, which took place in 1000 ns (with a dt equal to 0.002). The temperature was kept constant at 300 K and with a coupling constant (τT) equal to 0.1 ps. The Parrinello–Rahman coupling was used to keep the pressure at 1 bar. The isothermal compressibility was set at 4.5 × 10^−5^ bar^−1^ and the coupling constant (τP) was 1 ps. The long-range electrostatic contributions were calculated using the Ewald method (PME) [26]. The cut-off radius was set at 1.0 nm for both electrostatic and Van der Waals interactions. The length of all covalent bonds was limited by the LINCS algorithm [27]. The reference energy groups were the protein and the ligand. Finally, an analysis of the ligand–protein binding affinity was performed with Poisson–Boltzmann molecular mechanics, a method that allowed the calculation of the binding free energy of protein–ligand complexes [28]. All of the simulations were performed in quintuplicate.

#### 2.1.4. Methods of Analysis

The last phase involved the analysis of the trajectories. Snapshots of molecular dynamics simulations were produced using CHIMERAX and PyMOL [29,30]. Plots were prepared with XMGRACE software (http://plasmagate.weizmann.ac.il/Grace/, accessed on 11 July 2022).

Variation of the radius of gyration. The variation of the radius of gyration was an indicator of the compactness of the complex protein–ligand. It defined the root-mean-square distance of each atom (or monomer) from the center of mass.

Protein–ligand interaction and energy analysis. Protein–ligand interaction analysis was useful for determining which amino acids were involved in forming hydrogen bonds with the ligand. Therefore, the CHIMERAX software was used to visualize the Van der Waals contacts and the hydrogen interactions that were formed. The Gromacs *g_energy* tool was used to obtain the potential energy values.

RMSD and RMSF. The variation of the protein–ligand complex was determined by the root-mean-square deviation (RMSD) during the 10,000 ps MD simulation. The RMSD was used to measure the equilibration, protein flexibility, and the average distance between backbone atoms of a protein. It was employed to evaluate whether the simulated systems had reached an equilibrium phase. Root-mean-square deviation of atomic positions (RMSF) was used to establish the average measure of the distance between the atoms of molecules superimposed on each other. From the RMSF profile, it was possible to identify which amino acids were most involved in the binding site.

Free binding energy. The online script MMPBSA.py was used to perform the energy binding calculations according to the Poisson–Boltzmann equations.

### 2.2. In Vitro Methods

#### 2.2.1. Drugs and Chemicals

HA of low molecular weight (from 80 to 150 kDa) conjugated with lactose-derived residues (HYLACH^®^, Jointherapeutics, Como, Italy) and in the native state were dissolved in saline phosphate before use.

#### 2.2.2. Primary Human Bronchial Fibroblasts and U937 Monocytes

Primary human bronchial fibroblasts were obtained by the cultivation of bronchial biopsies performed on one patient, a current smoker (60 pack/years) with near-normal lung function, and used for a different protocol study. Details of the bronchoscopic procedure, cultivation methods, and ethical statements related to that procedure are reported in a previous study [31]. All experiments were conducted in triplicate using these bronchial fibroblasts from one patient.

The human monocyte U937 cell line was obtained from Thermo Scientific (Wilmington, DE, USA) and cultured in RPMI (Euroclone, Pero, Italy), 10% fetal bovine serum (FBS) (Gibco, ThermoFisher Waltham, MA, USA), and 1% antibiotics at 37 °C and 5% CO_2_.

#### 2.2.3. Activated U937 Monocyte Conditioned Medium

U937 monocytes were differentiated into macrophages with phorbol 12-myristate 13-acetate (PMA) (Sigma-Aldrich, St. Louis, MO, USA) at a concentration of 50 ng/mL for 48 h and, subsequently, treated with 1 μg/mL lipopolysaccharide (LPS, Sigma) for 1 h. Cells were then cultivated in complete RPMI for 24 h and the inflammatory-conditioned medium (CM) was collected, centrifuged, and filtered before being used to treat human bronchial fibroblast cultures. The differentiation of monocytes to macrophages was verified under an inverted phase-contrast microscope and confirmed with the protein expression of cytokines IL-1β and TNF-α (Scheme S1)**.**

#### 2.2.4. Evaluation of the Effects of HYLACH on Primary Human Bronchial Fibroblast Viability

To assess the effect of different HYLACH preparations on the viability of cultured primary human bronchial fibroblasts, confluent cells were seeded at a density of 1200 cells in 96-well plates and grown in standard culture conditions for 24 h, 3 days, and 6 days with 0.5 mg/mL HA or HYLACH. Cell proliferation was evaluated by the MTT test (3-4,5-dimethylthiazol-2-yl-2,5-diphenyl tetrazolium bromide), according to a modified Denizot method [32]. The values of absorbance were calculated using Infinite F200 (TECAN). Each experiment was performed in triplicate.

#### 2.2.5. Analysis of Anti-Inflammatory and Antioxidative Effects Induced by HYLACH

The effects of HYLACH with different lactosylation degrees on human bronchial fibroblasts obtained from smokers and exposed to U937 CM were evaluated at different time points through the analysis of ROS generation detection and by the analysis of proinflammatory molecule expressions.

- Detection of intracellular ROS generation. Human bronchial fibroblasts were seeded in 96-well plates with a density of 10,000 cells/well, allowed to adhere overnight, and then, exposed to activated U937 CM to induce intracellular ROS generation. Cells were then cultivated in the presence or absence of HA or HYLACH for 4 h. Subsequently, the medium was removed, the cells were rinsed twice with PBS and then loaded with 5 µM 2′,7′-dichlorodihydrofluorescein diacetate (H2DCFDA; Molecular Probes, ThermoFisher, Waltham, MA, USA) diluted in PBS for 1 h at 37 °C. H2DCFDA is a nonfluorescent probe that, in the presence of intracellular ROS, rapidly oxidizes to the fluorescent 2′,7′-dichlorofluorescein. The cells were washed three times with PBS before measuring fluorescence intensity with a microtiter plate reader (Infinite 2000, Tecan, Milan, Italy) at 485 and 535 nm for excitation and emission, respectively.
- Gene expression of inflammatory molecules. In primary human bronchial fibroblasts exposed or not to activated U937 CM and treated with HA and HYLACH for 4, 10, and 24 h, differences in the gene expression of IL-1β, IL-6, TNF-α, Gal-1, Gal-3, and MMP-3 were examined by qPCR. Total RNA was purified using TRIzol (Life Technologies, Carlsbad, CA, USA) following the manufacturer’s instructions and the Nanodrop 2000c spectrophotometer (Thermo Scientific) was used to evaluate RNA quality by measuring absorbance at 260/280 nm. To remove DNA from the samples, total RNA was treated for 15 min with DNAse I (Thermo Fisher). Five hundred ng of total RNA were reverse transcribed with oligo-dT and Superscript II (Life Technologies, Carlsbad, CA, USA) to generate cDNA. Gene expression levels of cytokines and MMP-3 were evaluated with a Rotor-Gene RG-3000A (QIAGEN, Hilden, Germany) using the Xpert fast SYBR (GRISP, Porto, Portugal). The expression of target genes was normalized to the endogenous levels of peptidylprolyl isomerase A (PPIA). The 2^ΔCt^ method, where dCt = Ct PPIA − Ct target gene, was used to assess gene expression. Table 1 contains a list of the primers used in the qPCR analysis.
- Enzyme-linked immunosorbent assay (ELISA). Supernatants were collected from primary human bronchial fibroblast cultures stimulated or not with activated U937 CM for 24 h and subsequently treated with HA and HYLACH. IL-1β, TNF-α, and MMP-3 were quantified using ELISA kits from R&D Systems (Minneapolis, MN, USA), Gal-1 with the ELISA Kit from Ray Biotech (Ray Biotech, Inc., Peachtree Corners, GA, USA), IL-6 with the human ELISA kit from Boster Bio (Pleasanton, TX, USA) and Gal-3 with Sino Biological (Sino Biological, Wayne, PA, USA) according to the suppliers’ protocols. Each experiment was carried out in triplicate.

#### 2.2.6. Statistical Analysis

A one-way analysis of variance (ANOVA) using Tukey’s post hoc Test for multiple comparisons was employed, along with an unpaired Student’s *t*-test, to conduct the statistical analysis with GraphPad Prism 7 (San Diego, CA, USA). The statistical significance was set at *p* < 0.05.

## 3. Results

### 3.1. In Silico Results

#### 3.1.1. HYLACH Compounds Show a Better Docking Score Than Nonfunctionalized HA

The MAESTRO-prepared protein and ligands were used as starting structures for the molecular docking phase. AutoDock predicts bound conformations and identifies the structure of the ligand that interacts most efficiently with the protein (Gal-3). The output of the docking simulations is the estimated ΔG (kcal/mol) with which the ligand binds to the receptor protein pocket. The best estimated ΔG0 for HA is equal to −8.238705 kcal/mol, while for lactose it is −9.608375 kcal/mol, and for the molecules with different amounts of lactose-derived residues named HYLACH, the average of the best poses was equal to −8.872238 kcal/mol. The molecular dynamics simulations were carried out using the top-scoring binding poses of HA, Lactose, and HYLACH generated during the molecular docking step.

#### 3.1.2. HYLACH with a Percentage of Lactose-Derived Residues of up to 30% and Lactose Represent the Best Ligands for Gal-3

Ligands exhibit a lactosylation ranging from 0% (HA only) to 100%. Ligands with similar levels of lactosylation (20% range) display comparable outcomes; therefore, they were combined.

**Figure 1:**
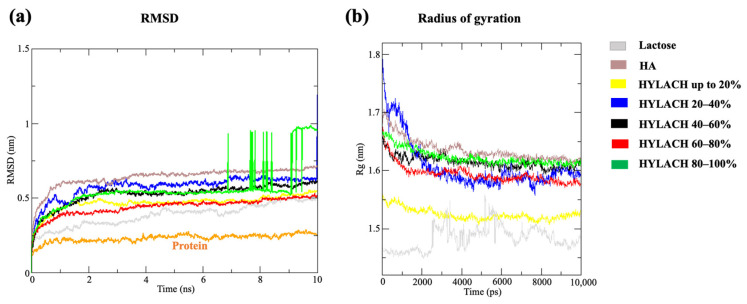
RMSD and Radius of gyration results. (**a**) The protein-associated RMSD curve is highlighted in orange and represents the most stable and equilibrated system, with an RMSD of 1.5 Å. Lactose, HYLACH of up to 20%, and HYLACH from 60 to 80% lactosylation were the systems that most closely resembled the unbound protein’s configuration. Rather, the system formed by Gal-3 and HA differed the most from the protein’s configuration. (**b**) The most structurally stable and compact system was the one containing lactose, even though the trend of the radius of gyration is not linear. Next, the most stable systems were Gal-3 and HYLACH of up to 20% and HYLACH of 20 to 40% lactosylation. The least compact was the protein–HA system with a radius of gyration of about 1.65 nm.

Systems are stable and compact within 2 ns from the start of the simulation. The stability of the protein–ligand complex was evaluated by considering the root-mean-squared deviation (RMSD) of the Cα atoms during the MD simulations (Figure 1a). Overall, after about 2 ns of simulations, the RMSD in each MD trajectory reached equilibrium, indicating that all studied systems were stable. The system containing the protein reached equilibrium after less than 1 ns, while the other systems containing HYLACH compounds, HA, and lactose, after about 2 ns. However, lactose, HYLACH with a percentage of lactose-derived residues of 60–80% and HYLACH of up to 20% lactosylation reached RMSD values very close to the value of the native structure of the protein. HA, on the other hand, had higher values of RMSD (5 Å); therefore, it is the protein–ligand complex that most deviated from the structure of the native protein (RMSD = 1.5 Å), indicating that the complex was unstable and the structure changed more prominently. It should be noted that HYLACH with 80–100% lactosylation had an RMSD that oscillated toward 6 ns from the beginning of the simulation; this demonstrated that the protein–ligand complex that formed became unstable at a certain point in the simulation. The analysis of the radius of gyration gave information on the degree of compactness of the protein–ligand complex. From the graphs (Figure 1b) it can be seen how the variation of the gyration radius for lactose, HYLACH of up to 20%, HYLACH 20–40%, and HYLACH from 60 to 80% lactosylation were much lower than for the other ligands. This indicates greater packing and better organization of complexes. Instead, the gyration radius associated with HA is higher, proving that the systems are structurally less stable.

**Figure 2:**
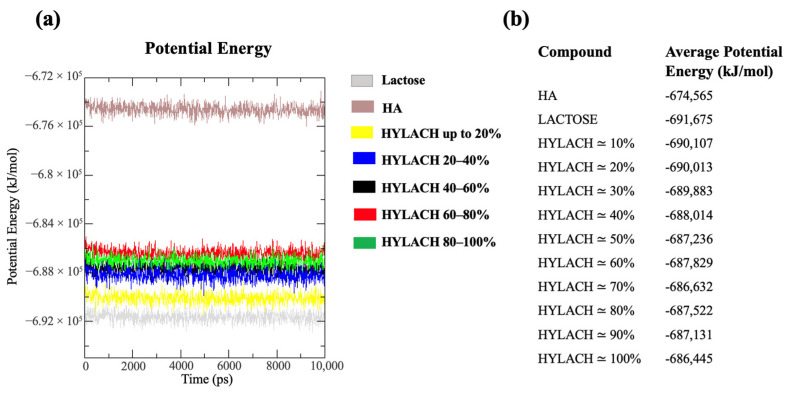
Results of potential energy. (**a**) The trend in simulation time (10,000 ps) of the potential energy of HYLACH, HA, and lactose and (**b**) Values of potential energy for lactose, HA, and percentages of HYLACH lactosylation. The lowest potential energy is associated with lactose and HYLACH of up to 30% of lactosylation. The system containing HA and Gal-3 is the least energetically favored as it has the highest potential energy value.

HYLACH compounds and lactose have lower potential energy and binding free energy than HA. The total potential energy was calculated by summing the contributions of Lennard–Jones, Coulomb, and the bonded terms. The analysis of potential energy shows that lactose had the lowest energy values, with an average potential energy value of −691,675 kJ/mol (Figure 2). The class of ligands with very low potential energy is represented by HYLACH of up to 20% and HYLACH of 30% lactosylation. HA had the highest potential energy; hence the protein–ligand complex is the most unstable from an energy perspective, with an average potential energy value of −674,565 kJ/mol. The average potential energy of each HYLACH compound considered is listed in the table (Figure 2b).

We also evaluated the binding free energies of the ligands. HA had the highest binding affinity value, probably due to repulsive interactions. The lowest energy value referred to HYLACH lactosylated up to 20%, indicating that it is the most energetically favored ligand. However, the other HYLACH compounds also exhibited very low binding energy values, indicating the formation of highly stable protein–ligand complexes from an energy viewpoint (Table 2).

**Figure 3:**
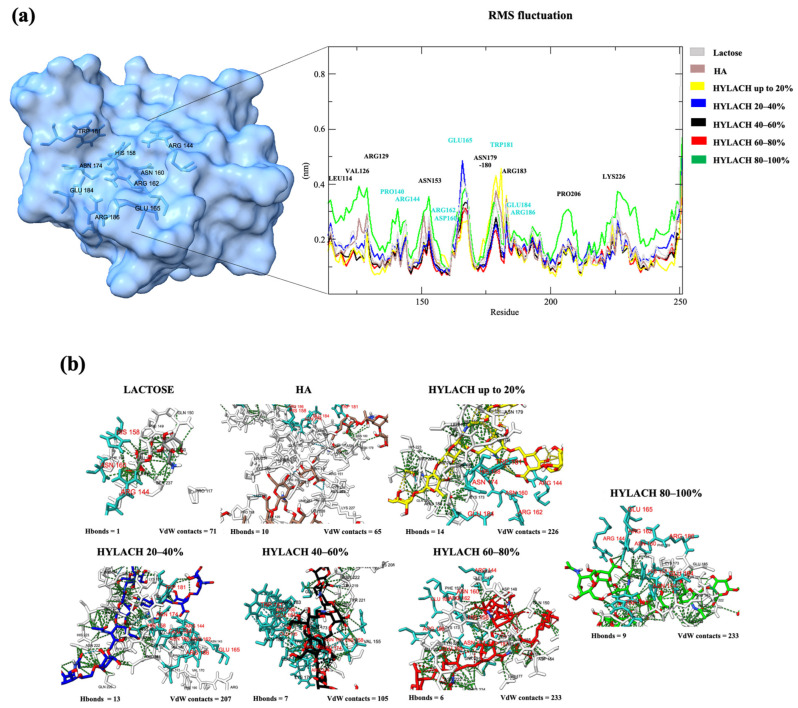
Interactions between Gal-3 and ligands. (**a**) RMS fluctuations show the amino acids most involved in the binding between the protein and the ligand. The peak indicates the presence of a very marked interaction. The changes in amino acids involved in the carbohydrate-recognition domain (amino acids highlighted in the figure) are extremely crucial. (**b**) Calculated interactions involving Gal-3 and ligands. The Van der Waals contacts between the receptor and the ligand are shown in green, while the intramolecular and intermolecular hydrogen interactions are shown in blue. The amino acids of the carbohydrate-binding domain are colored in cyan, while the other amino acids involved in the binding are white.

HYLACHs insert into the active site of Gal-3. The binding site of Gal-3 includes nine amino acids: TRP181, HIS158, ARG144, ASN160, ARG162, GLU165, ARG186, GLU184, and ASN174 [33]. The root mean square shows fluctuations at several amino acid levels, indicating an interaction between Gal-3 amino acids and the ligands. These fluctuations involve the residues associated with the CRD (Figure 3a), indicating that HA, HYLACH compounds, and lactose insert at the active binding site. Furthermore, it should be noted that mainly the molecule HYLACH up to 20% of lactosylation binds TRP 181 with an intense peak of fluctuation. The systems formed by Gal-3 and the following ligands produce the greater number of Van der Waals contacts: HYLACH up to 20% of lactosylation with 226 contacts, HYLACH 20–40% with 207 contacts, HYLACH between 60% and 100% with an average of 233 interactions. Regarding hydrogen interactions, both intra- and intermolecular interactions were considered. The better outcomes were as follows: HYLACH up to 20% and HYLACH 20–40% lactosylation with, respectively, an average of 14 and 13 hydrogen interactions. Lactose and HYLACH molecules fit perfectly into the carbohydrate-binding site, as shown in Figure 3b, while HA is orientated differently in the active site.

The in silico results show that nonfunctionalized HA represents the least effective ligand, as a very unstable Gal-3-HA complex is formed. The molecule with lactosylation degrees of up to 20% and with 20–40% of lactosylation were the best ligands for Gal-3 and the best potential energy and free binding energy values were obtained with HYLACH 10% and 30% lactosylation.

### 3.2. In Vitro Results

Following the results of the in silico studies, we chose to perform the in vitro experiments using HYLACH (Jointherapeutics, Como, Italy) molecules with a lactosylation degree of about 10% and 30%, hereinafter referred to as HYLACH 1 and HYLACH 2, respectively. We compared the anti-inflammatory and antioxidative effect of these molecules with that of a molecule with a higher percentage of lactosylation (of about 40%, HYLACH 3) and of the native HA from which the lactosylated molecule was obtained.

#### 3.2.1. HA and HYLACH Do Not Induce Changes in the Viability of Primary Human Bronchial Fibroblasts from Smokers

**Figure 4:**
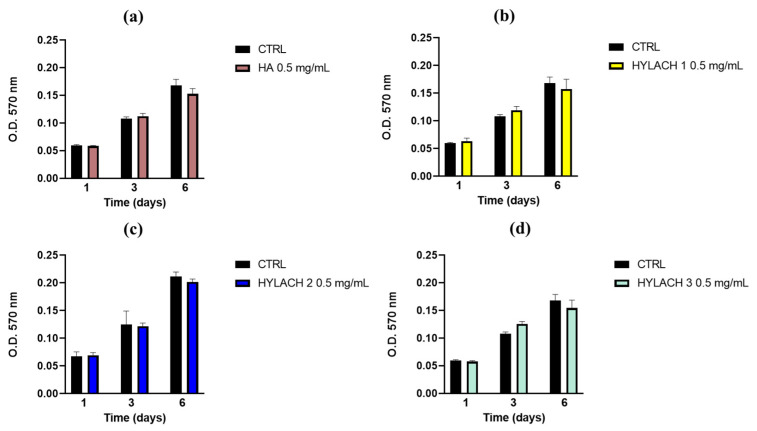
Time-dependent effects of HA and HYLACH on primary human bronchial fibroblast viability. Cells were seeded in 96-well culture dishes and exposed or not to HA (**a**), HYLACH 1 (**b**), HYLACH 2 (**c**), and HYLACH 3 (**d**) at the indicated concentration for various exposure times. The MTT test was performed 1, 3, and 6 days after treatment. Data are reported as mean ± SE of three independent experiments. Statistical differences based on unpaired Student’s *t*-test. Percentage of lactose-derived residues: HYLACH 1 = 10%, HYLACH 2 = 30%, HYLACH 3 = 40%.

In Figure 4, typical results relative to the viability of primary human bronchial fibroblasts cultivated in the presence of 0.5 mg/mL HA and HYLACH are reported. None of the tested molecules affected cell proliferation after 1, 3, and 6 days of treatment.

#### 3.2.2. HYLACH Exerts a Higher Anti-Inflammatory Effect Than HA on Primary Human Bronchial Fibroblasts

Activated U937 human monocytes can produce a wide variety of inflammatory molecules released in the conditioned medium (CM) mimicking a natural inflammatory event (Scheme S2).

**Figure 5:**
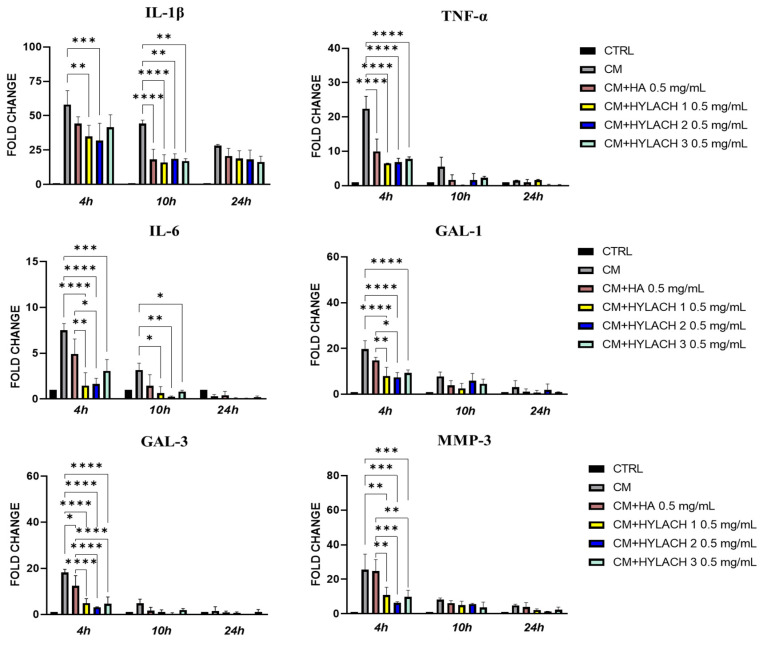
Bronchial fibroblast expression of proinflammatory and MMP molecules after exposure to U937 CM in the presence or absence of HYLACH with different degrees of lactosylation. Cells were exposed for 24 h to the CM of activated U937 and then cultured in the presence or absence of 0.5 mg/mL HA and HYLACH. RNA transcript levels specific for *IL-1β*, *TNF-α*, *IL-6*, *Gal-3*, *Gal-1*, and *MMP-3* were evaluated by qPCR, as described in the Materials and Methods section at 4, 10, and 24 h after treatment. Statistical differences are based on a one-way ANOVA test and data are expressed as mean ± SE obtained from three independent experiments. * *p* < 0.05 and ** *p* < 0.01; *** *p* < 0.001, and **** *p* < 0.0001. Percentage of lactose-derived residues: HYLACH 1 = 10%, HYLACH 2 = 30%, and HYLACH 3 = 40%.

When bronchial fibroblasts obtained from smokers were cultivated in the presence of the CM for 24 h, a significant upregulation of the proinflammatory cytokines *IL-1β*, *TNF-α*, *IL-6*, *galectins*, and *MMP-3* was found (Figure 5). Subsequently, HA, HYLACH 1, HYLACH 2, and HYLACH 3 compounds were administrated to the inflamed cell cultures for 4, 10, and 24 h, and qPCR analysis showed that all of the molecules reduced the cell expression of proinflammatory molecules *TNF-α* and *Gal-3* at 4 h, with the effect of HYLACHs being more significant than that of HA, especially toward *Gal-3* expression (*p* < 0.0001 vs. *p* < 0.05). Furthermore, HYLACH molecules, but not HA, also exerted a significant reduction in *IL-1β*, *IL-6*, *Gal-1*, and *MMP-3* at 4 h, and this effect was still evident at 10 h of culture. Of note, the inhibition of *IL-1β* and *IL-6* by HYLACH 1 and HYLACH 2 was higher than that exerted by HYLACH 3.

**Figure 6:**
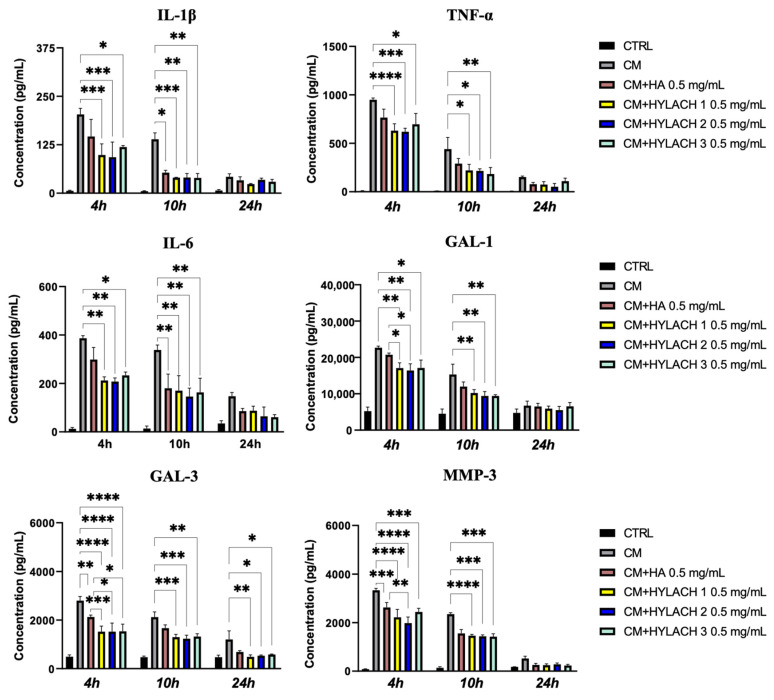
IL-1β, TNF-α, IL-6, Gal-1, Gal-3, and MMP-3 protein expression in primary bronchial fibroblasts after exposure to U937 CM in the presence or absence of HYLACH. Primary human smokers’ bronchial fibroblasts were cultivated in the presence or absence of 0.5 mg/mL HA and HYLACH after 24 h of exposure to activated U937 CM. At 4, 10, and 24 h following treatment, ELISA was used to analyze IL-1β, TNF-α, IL-6, Gal-1, Gal-3, and MMP-3 protein expressions. The one-way ANOVA test with multiple comparisons was used to determine statistical differences. The results are presented as mean ± SE of three independent experiments. CM stands for fibroblast cultures that have been exposed to U937 CM. * *p* < 0.05, ** *p* < 0.01, *** *p* < 0.001, and **** *p* < 0.0001 vs. respective CM-treated control. Percentage of lactose-derived residues: HYLACH 1 = 10%, HYLACH 2 = 30%, and HYLACH 3 = 40%.

The ELISA assays (Figure 6) confirmed that the CM of activated U937 human monocytes caused an increased protein expression of IL-1β, TNF-α, IL-6, Gal-1, Gal-3, and MMP-3 in cell cultures at 24 h after exposure. However, the supplementation of HA and HYLACH to the cell culture medium induced a significant downregulation of proinflammatory cytokine expression at the same time point after treatment, confirming the results obtained at the mRNA level. In fact, the downregulation of Gal-3 and MMP-3 was not only particularly marked (*p* < 0.0001) in the presence of HYLACH 2 but also significantly lower than that obtained in the presence of HA (*p* < 0.05). However, the decrease in Gal-3 expression induced by HYLACH 1 and HYLACH 2 was significantly higher (*p* < 0.0001) compared to the effect induced by HA (*p* < 0.001 and *p* < 0.05, respectively).

#### 3.2.3. HYLACH 1 and HYLACH 2 Exert an Antioxidant Effect on Primary Human Bronchial Fibroblasts Cultures Exposed to CM

**Figure 7:**
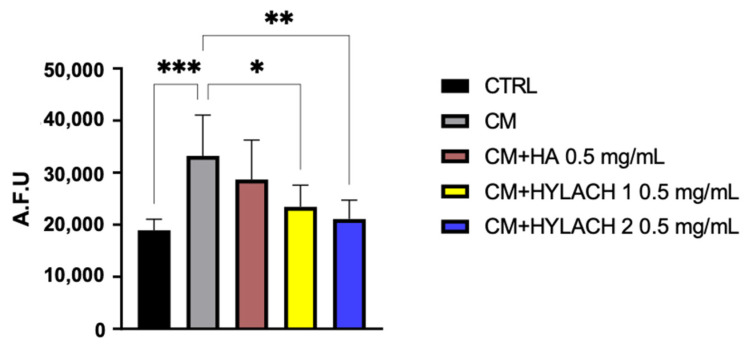
Reactive oxygen species (ROS) generated by primary human bronchial fibroblasts from smokers in the presence or absence of HYLACH with different degrees of lactosylation. Cells were cultured under optimal conditions and intracellular ROS generation was evaluated using CM for 12 h to induce intracellular ROS. Statistical differences are based on a one-way ANOVA test with multiple comparisons. Data are reported as mean ± SE of the percentage of fluorescence observed in at least two independent experiments. * *p* < 0.05, ** *p* < 0.01 and *** *p* < 0.001 compared with respective CM-treated control. Percentage of lactose-derived residues: HYLACH 1 = 10% and HYLACH 2 = 30%.

Intracellular ROS generation was evaluated using the H2DCFDA probe following cell exposure to CM of activated U937 monocytes (macrophages) for 12 h, in the presence or absence of HA, HYLACH 1, or HYLACH 2. As reported in Figure 7, the treatment of cell cultures with all of the tested HYLACH revealed a significantly decreased level of ROS (*p* < 0.05) when compared to the CM-treated human cell cultures, which was higher (*p* < 0.01) for HYLACH 2 (Figure 7). However, both of the tested HYLACH compounds revealed a significant antioxidative effect in comparison to CM and HA-treated cells.

## 4. Discussion

Cigarette smoke remains the main common risk factor for pulmonary diseases, such as COPD, and is associated with a greater risk of lung cancer [34]. The present study demonstrated that HYLACH^®^, a lactose-modified HA can bind to Gal-3 and exert anti-inflammatory and antioxidant effects when administered in vitro to the primary human bronchial fibroblasts of smokers exposed to the conditioned medium (CM) of activated U937 monocytes. The reduced inflammation is accompanied by a decrease in matrix protease expression, responsible for ECM degradation in many pulmonary diseases, such as COPD.

Our in silico results showed that HYLACH with a percentage of lactosylation of 10–40% is the best ligand for Gal-3 and the in vitro study demonstrated that the compounds HYLACH 1, HYLACH 2, and HYLACH 3 (10, 20, and 40% of lactosylation, respectively) restored gene and protein expression for *IL-1β*, *TNF-α*, *IL-6*, *Gal-1*, *Gal-3*, and *MMP-3* to near baseline values when administrated to cell cultures exposed to the CM of activated U937 cells. Moreover, we found that cells exposed to the CM underwent oxidative damage that was less pronounced when these cells were treated with HYLACH 1 and HYLACH 2 at the moment of in vitro injury. To the best of our knowledge, no previous data have been reported on the anti-inflammatory activity of HYLACH, so with this study, we compared the activity of HYLACH with that of HA for which the anti-inflammatory beneficial effect and protective action are well demonstrated [35]. The mixture of HA with other natural substances was also found to improve the therapeutic activity of HA [36,37], and recently, HA was also proposed in the treatment of airway diseases improving functional parameters in COPD patients [38]. The interrelationship between oxidative damage and inflammation is well demonstrated, and the role of HA in scavenging oxygen radicals has been recently reported [39,40].

It is also well demonstrated that Gal-3 is overexpressed by inflamed cells, sustaining the formation of a chronic inflammatory environment. In particular, Gal-3 was recently associated with inflammation and ECM degradation in COPD [10,41]. Moreover, we recently demonstrated that the combination of HA and lactose-modified chitosan can prolong and improve the well-known anti-inflammatory activity of HA, attenuating macrophage-induced inflammation, MMP expression, and ROS production [17,42]. Macrophages are crucial regulatory cells of host defense and macrophage-induced inflammation contributes to the injury of bronchial tissues [4,10,12,31]. We demonstrated that U937-activated monocytes can produce many proinflammatory molecules (unpublished data) that, in turn, induce cell production of inflammatory activators such as IL-1β, IL-6, TNF-α, and Gal-3. Therefore, targeting Gal-3 may be a beneficial strategy to inhibit bronchial inflammation.

Here, we used computational methods to study the interaction between Gal-3 and several ligands (HA, lactose, and HYLACH functionalized with different percentages of lactose) by targeting the protein carbohydrate-binding domain. The simulation results demonstrated that HA functionalized with lactosylation is a better ligand than lactose, as demonstrated by the free binding energy analysis and interaction analysis. However, nonfunctionalized HA represents the least effective ligand for the CRD binding domain of Gal-3 since a very unstable protein–ligand complex is formed. This finding could be due to the formation of repulsive interactions between the protein and the ligand. Furthermore, since it is not functionalized with lactose, which manages to enter the carbohydrate-binding site, the interaction with the protein is hindered. Moreover, RMSF and visualization analysis indicated that the residues of the CRD binding domain of Gal-3 are involved in the binding between the protein and the ligand. Briefly, considering the potential energy and radius of gyration analysis, the best ligands were lactose, HYLACH up to 20%, and HYLACH with a percentage of lactosylation from 20 to 40%. From the analysis of hydrogen and Van der Waals interactions, the best ligands were HA, HYLACH up to 20%, and HYLACH from 20 to 40% of lactosylation. Therefore, many Van der Waals interactions were formed by HYLACH with 60 to 80% lactosylation and from 80 to 100% lactosylation. The lower RMSD of the docking complexes are indications of overall systems stability and the most stable complexes were those formed by lactose, HYLACH from 60 to 80% lactosylation, and HYLACH up to 20% of lactosylation. Therefore, from these results, it emerged that the best ligand was HYLACH with a lactosylation between 10 and 30%. The results led to two HYLACH compounds (HYALCH 1 = 10% lactosylation and HYLACH 2 = 30% lactosylation). We, consequently, decided to test the anti-inflammatory and antioxidative properties of these two compounds in vitro and to compare their activity to that of HYLACH 3, which has a higher percentage of lactosylation. Since referential dosages for HYLACH are not reported, we chose to explore its performances at the concentration of 0.5 mg/mL based on HA literature suggestions showing a range of usefulness from 0.1 to 1 mg/mL HA. In particular, Cantor et al. found a therapeutic role of 0.01% aerosolized HA in COPD [43], whereas Brivio et al. demonstrated that the mixture of 0.1% HA and hypertonic saline exert a protective effect in reducing the inflammation of airways [44]. However, a US Food and Drug Administration review of the toxicology data submitted by Exhale Therapeutics (Belmont, CA, USA) advises not exceeding 0.1% HA solution (1 mg/mL) for COPD lung treatments, thus, a HYLACH concentration of 0.5 mg/mL could be considered a reasonable compromise to explore the performances of the tested compounds. We demonstrated that the treatment of primary human bronchial fibroblasts of smokers exposed to macrophage CM with 0,5 mg/mL HYALCH 1, 2, and 3 induces the downregulation not only of IL-1β, IL-6, and TNF-α gene expression but also of galectins, potent regulators of growth that bind to cell surface glycans with relevance for inflammation. This effect is presumably due to the interaction of galectins with the lactose residues of HYLACH compounds, which inhibits cell surface binding. We found also that HYLACH 1 and HYLACH 2 led to greater downregulation of IL-1β, IL-6, and Gal-3 compared to HYLACH 3, showing a clear anti-inflammatory effect. HYLACH compounds not only reduced the proinflammatory molecule gene expression but also accompanied the decrease in MMP-3 expression, and it is well documented that cytokines such as IL-1β, TNF-α, and Gal-3 not only suppress the synthesis of ECM but also significantly upregulate MMP gene expression [45], representing an important challenge factor for lung damage, counteracted by HYLACH activity. Furthermore, we found that bronchial fibroblasts exposed to the CM of activated U937 underwent oxidative damage that was less pronounced when these cells were treated with HYLACH 1 and HYLACH 2, if compared to HA, showing the capability of developing an antioxidant activity. However, the specific mechanisms for the anti-inflammatory and antioxidant activity developed by HYLACH 1 and 2 need to be specifically investigated. This will be a matter of the next study in our group. Moreover, to increase the opportunities for managing chronic diseases, it would also be of fundamental importance to develop HYLACH-based biodegradable and clearable inorganic nanomaterials for biomedical applications [46].

In conclusion, our study demonstrated that HYLACH 1 and HYLACH 2 are able not only to neutralize the upregulation of inflammatory cytokines and galectins but also to strongly decrease MMP-3 expression and oxidation in our primary bronchial fibroblasts from smokers with a near-normal lung function. These data may be relevant in terms of the prevention of pulmonary deterioration and progression of diseases and may be considered as an initial step in the development of new therapeutic treatments highly needed for these chronic smoking-related pulmonary diseases.

## Figures and Tables

**Table 1:** Genes investigated and primers used.

Gene (Accession Number)	Name	Primer Sequences
*IL-1β* (NM_000576.3)	*Interleukin 1 beta*	Fw 5′-GAATCTCCGACCACCACTACAG-3′ Rv 5′-TGATCGTACAGGTGCATCGTG-3′
*LSGALS1* (NM_002305.4)	*Galectin 1*	Fw 5′-TCTCGGGTGGAGTCTTCTGA-3′ Rv 5′-GTTCAGCACGAAGCTCTTAGC-3′
*LSGALS3* (NM_002306.4)	*Galectin 3*	Fw 5′-CTGCTGGGGCACTGATTGT-3′ Rv 5′-TGTTTGCATTGGGCTTCACC-3′
*MMP-3* (NM_002422.5)	*Matrix metalloproteinase 3*	Fw 5′-TCACTCACAGACCTGACTCG -3′ Rv 5′-AAAGCAGGATCACAGTTGGC-3′
*IL-6* (NM_000501.4)	*Interleukin 6*	Fw 5′-ATGAACTCCTTCTCCACAAGCG-3′ Rv 5′-CTCCTTTCTCAGGGCTGAG-3′
*PPIA* (NM_021130.5)	*Peptidylprolyl Isomerase A*	Fw 5′-GGGCTTTAGGCTGTAGGTCAA-3′ Rv 5′-AACCAAAGCTAGGGAGAGGC-3′
*TNF-α* (NM_000594.3)	*TNF-alpha*	Fw 5′-AAGCCTGTAGCCCATGTTGT-3′ Rv 5′-GGACCTGGGAGTAGATGAGGT-3′

Fw = forward; Rv = reverse.

**Table 2:** Average binding free energies of the ligands.

Compound	% of Lactosylation	Average Binding Free Energy (kJ/mol)
HA	-	4138
LACTOSE	-	−39
HYLACH	10%	−302
	20%	−236
	30%	−284
	40%	−87
	50%	−166
	60%	−197
	70%	−218
	80%	−222
	90%	−212
	100%	−282

## Data Availability

The data presented in this study are available on request from the corresponding author.

## Supplementary Materials

The following supporting information can be downloaded at: https://www.mdpi.com/article/10.3390/polym15071616/s1. Scheme S1: Detection of IL-1β and TNF-a in activated U937 human monocytes; Scheme S2: Inflammatory molecules expression by activated U937 cells (ELISA).
