# Supplementary material for: The Anti-Inflammatory Effect of Lactose-Modified Hyaluronic Acid Molecules on Primary Bronchial Fibroblasts of Smokers
Source: Polymers (Basel). 2023 Mar 24;15(7):1616. doi: 10.3390/polym15071616 (PMC10096707; doi:10.3390/polym15071616)
Supplement: Supplementary file 1 [file polymers-15-01616-s001.zip › polymers-2216077-supplementary.pdf]

Supplementary Materials

The Anti-Inflammatory Effect of Lactose-Modified Hyaluronic Acid Molecules on Primary Bronchial Fibroblasts of Smokers

Alice Donato [1], Federico Fontana [2], Rina Venerando [3], Antonino Di Stefano [4] and Paola Brun [1,*]

[1] Histology Unit, Department of Molecular Medicine, University of Padova, 35121 Padova, Italy
[2] Center for Nanomedicine and Tissue Engineering, A.S.S.T. Grande Ospedale Metropolitano Niguarda, Piazza dell'Ospedale Maggiore 3, 20162 Milan, Italy
[3] Department of Molecular Medicine, University of Padova, 3521 Padova, Italy
[4] Divisione di Pneumologia e Laboratorio di Citoimmunopatologia dell'Apparato Cardio Respiratorio, Istituti Clinici Scientifici Maugeri, IRCCS, 28013 Gattico-Veruno (NO), Italy
* Correspondence: paola.brun@unipd.it

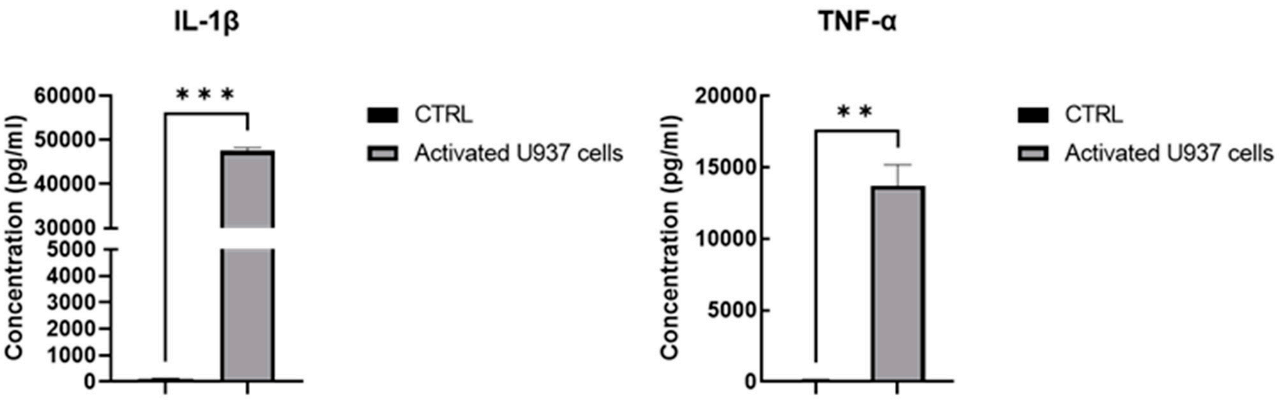

Scheme S1. Detection of IL-1β and TNF-*α* in activated U937 human monocytes. After 48 hours of PMA and 1 hour of LPS treatment, protein expression of IL-1β and TNF-*α* cytokines was measured using ELISA after 24 hours. The data are presented as mean ± SE of three independent experiments. The unpaired Student's t-test is used to calculate statistical differences. $^{**}P<0.01$, $^{***}P<0.001$ when compared to untreated cells.

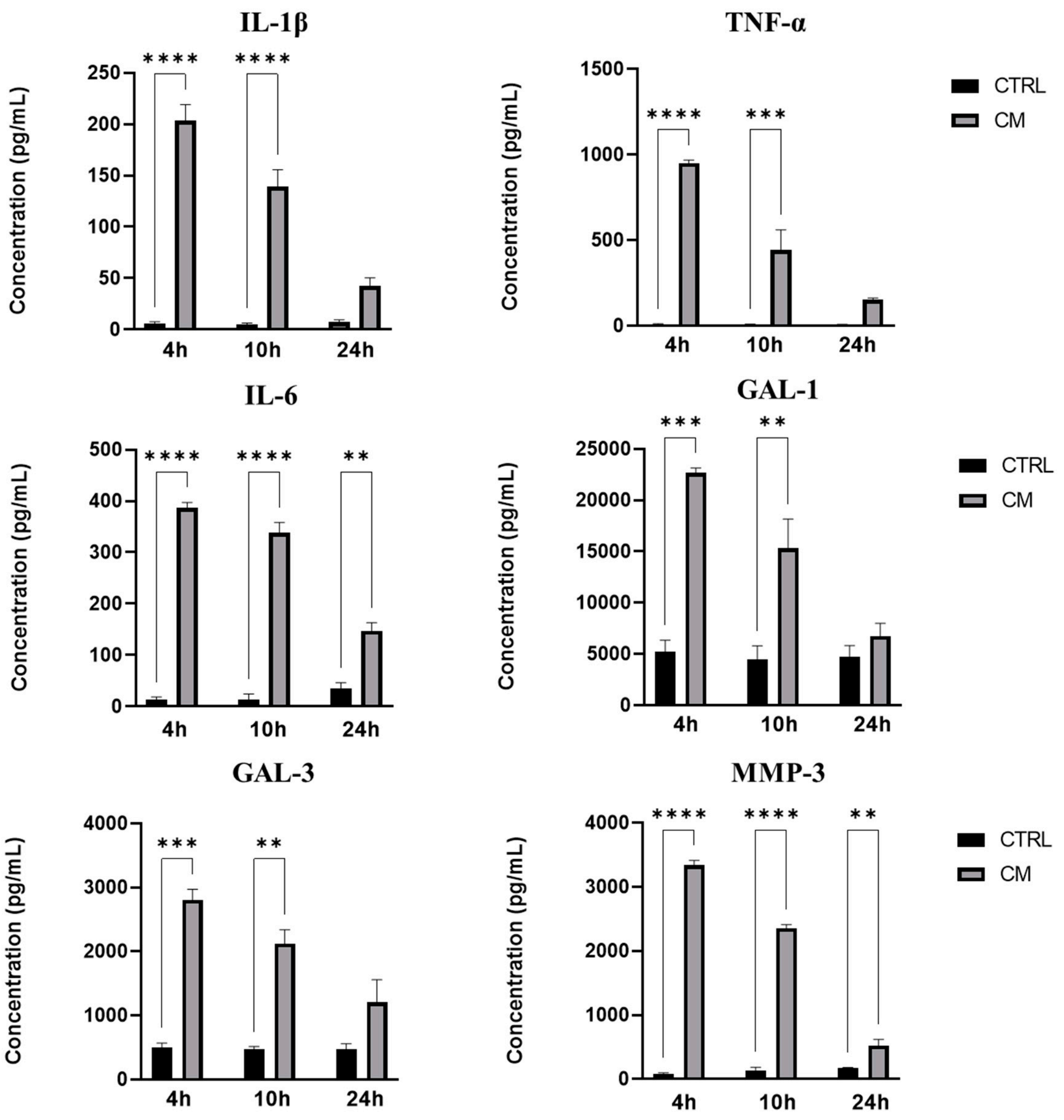

Scheme S2. Inflammatory molecules expression by activated U937 cells (ELISA). After 24h of exposure to CM of activated U937, primary human bronchial fibroblasts were *in vitro* cultivated for 4, 10 and 24 h. Pro-inflammatory molecules, galectins and MMP-3 protein expression were tested by ELISA analysis. Data from three independent experiments are presented as mean standard error (SE). Statistical differences based on unpaired Student's t-test. ** $p < 0.01$, *** $p < 0.001$ and **** $p < 0.0001$.
